# A new case management concept to decrease the rehospitalisation rate in heurischemic

**DOI:** 10.1186/1753-6561-5-S6-P326

**Published:** 2011-06-29

**Authors:** G Rümenapf, S Geiger, A Godel, W Vogelsang, S Morbach, N Wilhelm, N Nagel

**Affiliations:** 1 Clinic for Vascular Surgery, Diakonissen-Stiftungs-Krankenhaus Speyer , Speyer, Germany; 2 Case Management, Diakonissen-Stiftungs-Krankenhaus Speyer , Speyer, Germany; 3 Medical Controlling, Diakonissen-Stiftungs-Krankenhaus Speyer , Speyer, Germany; 4 CEO, Diakonissen-Stiftungs-Krankenhaus Speyer , Speyer, Germany; 5 Dept. Of Diabetology and Angiology, Marienkrankenhaus Soest, Soest, Germany; 6 Konzeptmanagement, B. Braun Melsungen AG, Melsungen, Germany; 7 Medical Scientific Affairs OPM, B. Braun Melsungen AG, Melsungen, Germany

## Introduction / objectives

The treatment of patients suffering from the neuroischemic diabetic foot syndrome (DFS) comprises arterial revascularization (e.g. below-knee bypass surgery), minor amputations, debridements, as well as long-term specialized wound care. Following premature discharge to the homecare sector, the quality of postoperative care is often inadequate. Many patients are readmitted. We studied the influence of a trans-sectoral case management (CM), ensuring outpatient care according to our clinical standards, on the readmission rate, length of hospital stay (LOS) and the hospital's costs/benefit situation.

## Methods

DFS patients after implementation of the CM (Case Management Group (CMG); n = 202; 2007-2008) were compared with a historic control group (HCG; n = 190; 2005-2006). All patients had high maintenance foot wounds as well as healing incisional wounds following bypass surgery. Both groups were matched for the principal diagnosis, a patients clinical complexity level (PCCL) of 4, and G-DRG-related flat rate. From the 202 CMG patients evaluated, 54 received long-term trans-sectoral care by the CM.

## Results

The rehospitalization rate in the CMG was significantly reduced versus the HCG (9,8 % vs.16,7%; p = 0,041). The reduction of the revolving door effect in the CMG significantly improved the costs/revenue situation for the hospital. The LOS was unchanged.

## Conclusion

The implementation of a hospital-based trans-sectoral CM significantly reduces the rehospitalization rate in patients with neuroischemic DFS requiring bypass surgery. Hospital economics are improved.

## Disclosure of interest

None declared.

